# Navigating the future of genitourinary cancer treatment: a decade of progress in image-guided surgery

**DOI:** 10.1097/JS9.0000000000001438

**Published:** 2024-04-09

**Authors:** Ying Liu, Aimin Jiang, Jinxin Li, Ziwei He, Peng Luo, Le Qu, Linhui Wang

**Affiliations:** a Department of Urology, Changhai Hospital, Naval Medical University, Shanghai; b Department of Oncology, Zhujiang Hospital, Southern Medical University, Guangzhou; c Department of Urology, Affiliated Jinling Hospital, Medical School of Nanjing University, Nanjing, People’s Republic of China

*Dear Editor,*

In the past decade, the realm of prostate cancer (PCa) treatment has witnessed a transformative shift toward precision medicine, epitomized by the integration of image-guided surgery (IGS). A recent comprehensive bibliometric study in the *International Journal of Surgery* titled ‘Knowledge mapping of application of image-guided surgery in prostate cancer: a bibliometric analysis (2013–2023)’ by Zeng and colleagues encapsulates this evolution, marking the first of its kind to holistically chart the landscape of IGS in PCa^1^. This study not only illuminates the prolific contributions spanning 75 countries and 2883 institutions but also underscores the United States’ pivotal role in steering global research efforts. This study not only offers a panoramic view of the current state of IGS in PCa but also provides a beacon for future research directions, emphasizing the critical role of PSMA (prostate-specific membrane antigen) and PET/CT (positron emission tomography/computed tomography) as enduring research hotspots. As we stand on the cusp of revolutionizing PCa treatment, this study serves as both a testament to our journey thus far and a compass for navigating the uncharted waters ahead. In addition to its widespread application in the treatment of PCa, the IGS system has also made significant strides in other urological cancers, including renal and bladder cancers.

The utilization of IGS has significantly advanced the field of kidney surgeries, specifically in nephrectomy and partial nephrectomy procedures, through enhancements in tumor localization, delineation of tumor margins, and preservation of renal function. The incorporation of real-time ultrasound, CT, and MRI imaging technologies has empowered surgeons with intricate anatomical insights, facilitating precise tumor excision and maximal preservation of renal tissue. Recent innovations, such as the integration of three-dimensional (3D) imaging and augmented reality (AR), have further enriched surgical planning and execution by providing surgeons with a holographic representation of the kidney’s anatomy and pathology. Prior studies have demonstrated the utility of 3D static and elastic AR systems that superimpose in-vivo anatomy during robot-assisted partial nephrectomy for complex tumors in aiding the identification of lesions and intraparenchymal structures that may be challenging to visualize with ultrasound alone. This has the potential to enhance the precision of the resection process and decrease postoperative complications, leading to improved functional recovery^2,3^.

Bladder surgeries, such as tumor resections and cystectomies, have experienced improvements in the visualization of tumors and accuracy of resection margins through the utilization of IGS. Fluorescence-guided surgery, employing agents like indocyanine green (ICG), has demonstrated potential in real-time identification of cancerous tissue within the bladder, enabling more comprehensive and precise tumor removal. A study conducted by Bryan *et al*. demonstrated that the utilization of a visual scoring system, such as a ‘Likert scale,’ can aid in the identification of non-invasive bladder tumors while performing a biopsy during the initial diagnostic flexible cystoscopy in an outpatient setting and is beneficial for confirming the presence of bladder cancer^4^. Furthermore, the incorporation of IGS with endoscopic techniques has facilitated minimally invasive strategies in bladder surgery, leading to decreased recovery periods and enhanced surgical results.

In the field of prostate surgery, specifically prostatectomy, IGS has played a crucial role in preserving nerve function and decreasing post-operative complications like incontinence and erectile dysfunction. The integration of MRI and ultrasound fusion imaging has become essential in identifying and protecting the neurovascular bundles adjacent to the prostate. Additionally, the introduction of robotic-assisted laparoscopic prostatectomy (RALP) in conjunction with IGS methods has heightened the accuracy of prostate tissue removal, ultimately enhancing patient results. Porpiglia and colleagues demonstrated that the implementation of elastic 3D virtual models accurately simulates prostate deformation during surgery, enabling correct identification of lesion location in dynamic reality. This may lead to a potential reduction in positive surgical margin rates and optimization of functional outcomes^5^.

The advancement of IGS in urology is contingent upon the enhanced incorporation of imaging technologies, robotics, and artificial intelligence (AI). Progress in imaging modalities such as PET/MRI fusion and optical coherence tomography (OCT) is anticipated to yield heightened resolution and contrast, facilitating the early detection of malignant tissues. The evolution of AI algorithms capable of real-time interpretation of imaging data stands to enhance surgical decision-making by potentially forecasting tissue properties and directing surgical procedures.

Furthermore, advancements in robotic surgery platforms are expected to further improve the accuracy and adaptability of IGS, enabling the performance of increasingly intricate and minimally invasive urological procedures. Additionally, the utilization of nanotechnology in imaging agents holds promise for revolutionizing the visualization of minute pathological processes, potentially facilitating highly precise surgical interventions. Concurrently, there is an increasing focus on individualized surgical planning and simulation for patients. The advancement of virtual reality (VR) and AR technologies is facilitating the creation of personalized 3D models of urological anatomy, allowing surgeons to strategize and practice procedures in a simulated setting. This methodology holds the potential to reduce surgical complications and enhance overall surgical results.

In summary, IGS has demonstrated notable advancements in enhancing the accuracy and safety of genitourinary surgical procedures within the field of urology. Through the utilization of sophisticated imaging modalities, surgeons are able to execute minimally invasive and precise surgical interventions, resulting in improved patient outcomes. Future prospects include the incorporation of AI, robotic assistance, and customized surgical planning, which have the potential to further transform urological surgery by enhancing safety, efficacy, and individualized patient care. As the field continues to evolve, ongoing research and innovation will be critical in unlocking the full potential of IGS in urology.

## Ethical approval

Not applicable.

## Consent

Not applicable.

## Sources of funding

Not applicable.

## Author contribution

Y.L., A.J., J.L., Z.H., and P.L.: data collection and manuscript preparation; L.Q. and L.W.: study concept or design and critical revision and submission.

## Conflicts of interest disclosure

There are no conflicts of interest.

## Research registration unique identifying number (UIN)

This is a commentary.

## Guarantor

Linhui Wang and Le Qu.

## Data availability statement

The datasets generated and/or analyzed during the current study are available from the corresponding author on reasonable request.

## Provenance and peer review

Not available.

## References

[1] Zeng N Sun J-X Liu C-Q . Knowledge mapping of application of image-guided surgery in prostate cancer: a bibliometric analysis (2013–2023). Int J Surg 2023;4. [Epub ahead of print]. doi:10.1097/JS9.0000000000001232 PMC11093506 38445538

[2] Porpiglia F Checcucci E Amparore D . Three-dimensional augmented reality robot-assisted partial nephrectomy in case of complex tumours (PADUA ≥10): a new intraoperative tool overcoming the ultrasound guidance. Eur Urol 2020;78:229–38. 31898992 10.1016/j.eururo.2019.11.024

[3] Zhu G Xin JC Weng GB . Application of holographic image navigation in urological laparoscopic and robotic surgery. Chin J Urol 2020;41:131–7.

[4] Bryan RT Liu W Pirrie SJ . Comparing an imaging-guided pathway with the standard pathway for staging muscle-invasive bladder cancer: preliminary data from the BladderPath study. Eur Urol 2021;80:12–5. 33653635 10.1016/j.eururo.2021.02.021

[5] Porpiglia F Checcucci E Amparore D . Three-dimensional elastic augmented-reality robot-assisted radical prostatectomy using hyperaccuracy three-dimensional reconstruction technology: a step further in the identification of capsular involvement. Eur Urol 2019;76:505–14. 30979636 10.1016/j.eururo.2019.03.037

